# Double plasma molecular adsorption system combined with plasma exchange successfully treated thyroid storm and severe liver injury: A case report

**DOI:** 10.1097/MD.0000000000046686

**Published:** 2025-12-19

**Authors:** Jiali Liu, Weiwei Zhang

**Affiliations:** a Internal Medicine Nursing Teaching and Research Department, Chongqing Medical and Pharmaceutical College, Chongqing, China; b Department of Endocrinology and Metabolism, The Second Affiliated Hospital of Chongqing Medical University, Chongqing, China.

**Keywords:** DPMAS, Graves’ disease, liver injury, plasma exchange, radioactive iodine treatment

## Abstract

**Rationale:**

Graves’ disease is the most common cause of hyperthyroidism, and antithyroid drugs are the main treatment, but they have the side effect of liver injury. Uncontrolled hyperthyroidism can trigger a life-threatening thyroid storm.

**Patients concerns:**

A 15-year-old girl developed severe liver injury and mononucleosis caused by Epstein-Barr virus infection after methimazole treatment, which eventually induced thyroid storm.

**Diagnoses:**

Graves’ disease, liver injury, mononucleosis, Epstein-Barr virus infection, thyroid storm.

**Interventions:**

Conventional medical treatment was ineffective, and the patient finally received the double plasma molecular adsorption system (DPMAS) combined with plasma exchange therapy and radioactive iodine treatment.

**Outcomes:**

The patient’s critical condition was relieved after 2 courses of DPMAS combined with plasma exchange therapy, and hyperthyroidism was controlled after radioactive iodine treatment.

**Lessons:**

DPMAS combined with plasma exchange therapy is effective in controlling thyroid storm with severe liver injury. It is also important for such patients to choose the appropriate time for radioactive iodine therapy.

## 1. Introduction

Hyperthyroidism is a disorder characterized by excessive synthesis and secretion of thyroid hormones, of which Graves’ disease is the commonest cause. Thyroid stimulating hormone receptor antibodies, also known as TRAb, can stimulate the thyroid gland and is the pathogeny of Graves’ disease.^[1]^ The main treatment options for Graves’ disease include antithyroid drugs (ATDs) and radioactive iodine.^[2]^ In some regions, ATDs are the first choice. ATDs include imidazoles and thiouracils, with methimazole and propylthiouracil (PTU) as representatives, and the 2 have some similarities in their side effects.^[3]^ Among the side effects of ATDs, liver injury, and granulocytopenia even sometimes leading to agranulocytosis, are of particular concern to clinicians. Radioactive iodine therapy is recommended for patients with Graves’ disease who experience severe drug side effects during ATDs treatment. Uncontrolled hyperthyroidism can lead to thyroid storm due to triggers such as infection, surgery, and trauma. Thyroid storm is a serious life-threatening condition with a mortality rate of 20% to 30%.^[4]^

This case reports a patient with Graves’ disease who suffered severe liver injury after taking methimazole, accompanied mononucleosis by Epstein-Barr virus (EBV) infection, which eventually induced thyroid storm. Her condition was extremely critical, and eventually well controlled through double plasma molecular adsorption system (DPMAS) combined with plasma exchange and radioactive iodine therapy.

## 2. Case presentation

A 15-year-old girl was admitted to hospital with a sore throat and fever for 4 days, and yellowing of her skin and eyes for 1 day. Two months prior, she was diagnosed with Graves’ disease due to high thyroid hormone levels, low thyroid stimulating hormone level, and significantly elevated TRAb level. At that time, her liver function and blood routine tests showed no abnormalities, and she was treated with 20 mg of methimazole orally daily. Before admission, she had been taking methimazole for more than 50 days and had not undergone a follow-up test. Her physical examination showed T 39.3°C, HR 133 beats/min, R 22 beats/min, BP 89/61 mmHg, and BMI 17.0 kg/m^2^. She was irritable and sweaty, with mild exophthalmos and an enlarged thyroid gland. In addition to yellowing of the skin and sclera, her pharynx was congested, and enlarged lymph nodes were palpable in her neck.

Her blood routine test revealed leukocytes 9.91 × 10^9/L (normal value 3.5-9.5 × 10^9/L), lymphocyte percentage 50% (normal value 20–40%), and manual testing found atypical lymphocytes. A bone marrow smear also found 11% atypical lymphocytes. The pharyngeal swab tested positive for EBV nucleic acid. The results of EBV-related antibody test indicated that she had acute EBV infection: viral capsid antigen (VCA)-IgM 136 U/ml (normal value<40 U/ml), VCA-IgG 108 U/ml (normal value <20 U/ml), early antigen (EA)-IgG 5.1 U/ml (normal value<10 U/ml), and Epstein-Barr nuclear antigen (EBNA)-IgG 4.0 U/ml (normal value <20 U/ml). Liver function tests showed alanine aminotransferase (ALT) 80U/L (normal value 7–40 U/L), aspartate aminotransferase (AST) 109 U/L (normal value 13–35 U/L), alkaline phosphatase (ALP) 228 U/L (normal value 35–100 U/L), gamma-glutamyl transferase (GGT) 141 U/L (normal value 7–45 U/L), total bilirubin (TBIL) 277.0 µmol/L (normal value 5.1–28.0 µmol/L), direct bilirubin 185.6 µmol/L (normal value 0.0–10.0 µmol/L), indirect bilirubin 91.4 µmol/L (normal value 1.5–18.0 µmol/L), and total bile acid (TBA) 197.5 umol/L (normal value 0.0–10.0 µmol/L). Coagulation parameters found activated partial thromboplastin time (APTT) 45.7 s (normal value 31.5–43.5 s), fibrinogen degradation product 5.83 μg/ml (normal value 0.01–5.00 μg/ml), prothrombin time (PT) 14.2 s (normal value 11.0–14.5 s), and international normalized ratio 1.09 (normal value 0.70–1.30). Her D-dimer was 1069.3 ng/ml (normal value 0–550.0 ng/ml). Abdominal color Doppler ultrasonography showed hepatosplenomegaly. Results of tests for hepatotropic virus and autoimmune hepatitis were unremarkable. Her thyroid tests revealed thyroid stimulating hormone < 0.005 uIU/ml (normal value 0.35–5.00 uIU/ml), free triiodothyronine (FT3) 29.6 pmol/L (normal value 3.1–6.8 pmol/L), free thyroid hormone (FT4) 90.3 pmol/L (normal value 9.5–24.5 pmol/L), TRAb 30.54 IU/ml (normal value 0.00–1.75 IU/ml). Her thyroid iodine uptake was 70.7% at 6 hours and 69.4% at 24 hours (normal values 7–40% at 6h and 17–60% at 24 hours). A thyroid scan showed an enlarged thyroid gland with a rich blood supply. She also had elevated levels of inflammatory markers, including interleukin-2 receptor > 7500 U/ml (normal value 223–710 U/ml), interleukin-8 76.00 pg/ml (normal value 0.00–62.00 pg/ml), interleukin-10 54.70 pg/ml (normal value 0.00–9.10 pg/ml), Tumor necrosis factor alpha (TNF-α) 50.70 pg/ml (normal value 0.00–8.10pg/ml).

Based on her clinical symptoms and the above test results, she was diagnosed with infectious mononucleosis caused by EBV infection and severe liver injury. According to the common clinical assessment tool for thyroid storm, the Burch Wartofsky Point Scale, her score is 80 (Table 1), indicating that she also suffered from thyroid storm. Poorly controlled hyperthyroidism led to decreased immunity, which may have been the cause of her EBV infection. Her liver injury was most likely attributable to the side effect of methimazole, although EBV infection may also exacerbate liver injury. Her uncontrolled thyroid hormone levels, combined with the infection and liver injury, triggered a thyroid storm.

**Table 1:** Assessment of thyroid storm based on the Burch Wartofsky Point Scale.

Criteria		Points	Patient condition	Patient score
Temperature (℃)	37.2–37.7	5	39.3℃	20
	37.8–38.3	10		
	38.4–38.8	15		
	38.9–39.4	20		
	39.5–39.9	25		
	≥40	30		
Heart rate (beats/min)	100–109	5	133/min	20
	110–119	10		
	120–129	15		
	130–139	20		
	≥140	25		
Atrial fibrillation	Absent	0	Absent	0
	Present	10		
Congestive heart failure	Absent	0	Absent	0
	Mild (pedal edema)	5		
	Moderate (bibasilar rales)	10		
	Severe (pulmonary edema)	15		
Gastrointestinal-hepatic dysfunction	Absent	0	Jaundice	20
	Moderate (diarrhea, nausea/vomiting, abdominal pain)	10		
	Severe (jaundice)	20		
Central nervous system effects	Absent	0	Agitation	10
	Mild (agitation)	10		
	Moderate (delirium, psychosis, extreme lethargy)	20		
	Severe (seizures, coma)	30		
Precipitating event	Absent	0	Present	10
	Present	10		
Total points				80

The total points < 25 points, unlikely to represent thyroid storm; 25–44 points, suggestive of mpending thyroid storm; ≥45 points, highly suggestive of thyroid storm.

**Figure 1.**
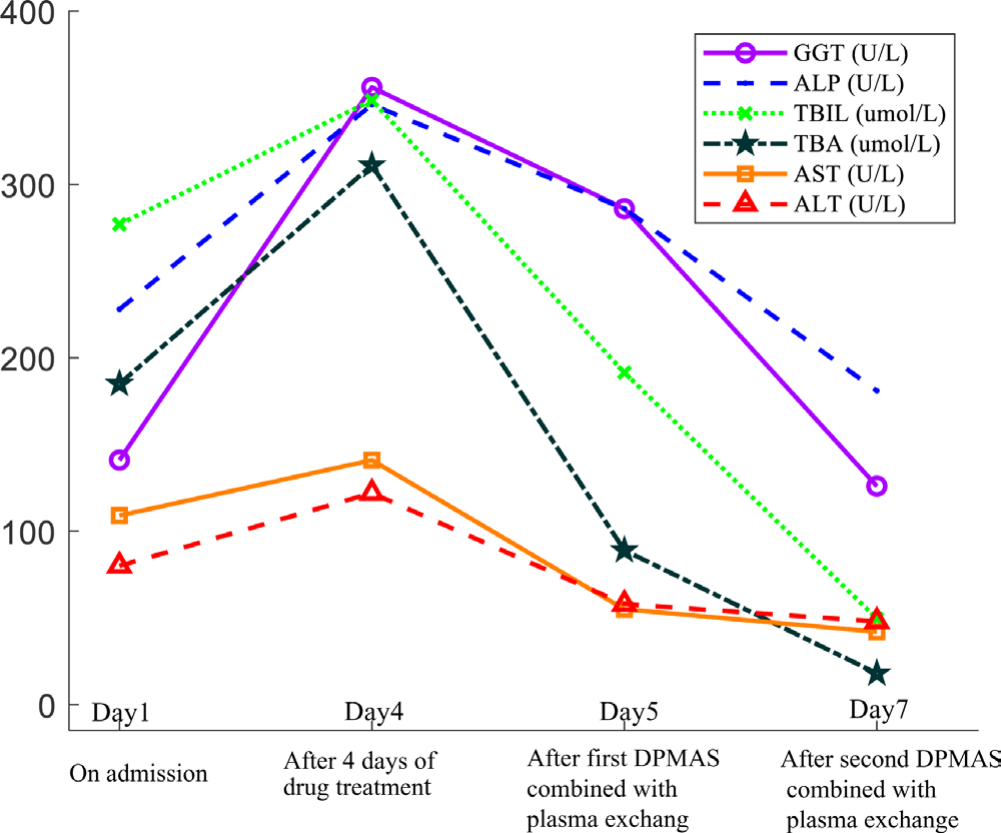
Changes in liver function of the patient after drug treatment and 2 courses of DPMAS combined with plasma exchange therapy. DPMAS = double plasma molecular adsorption system.

**Figure 2.**
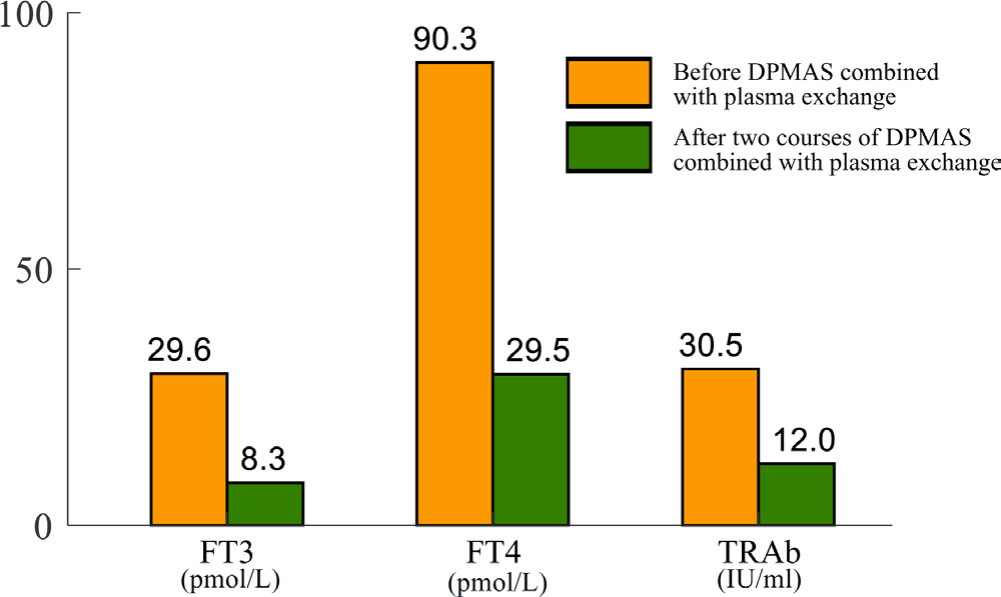
Changes of thyroid hormone and TRAb in the patient after 2 courses of DPMAS combined with plasma exchange therapy. DPMAS = double plasma molecular adsorption system.

Due to the complexity and severity of her condition, she underwent evaluation and treatment by a multidisciplinary team involving physicians from infectious diseases, endocrinology, and nuclear medicine. Initially, she discontinued methimazole and was administered propranolol, hydrocortisone, ganciclovir, and some liver-protective drugs including S-adenosy-L-methionine, polyenylphosphatidylcholine, and reduced glutathione. However, after 4 days of drug treatment, her condition did not improve, and her liver function further deteriorated, as evidenced by continued increases in transaminase, bilirubin, and bile acid levels (Fig. 1). Only the PT and international normalized ratio remained normal. Subsequently, in order to control her condition quickly, on the 5th and 7th day of hospitalization, she underwent DPMAS combined with plasma exchange therapy respectively, with 2000 ml of fresh plasma exchanged each time. Thus, her condition was controlled, with great recovery in liver function (Fig. 1), significant decreases in thyroid hormone and TRAb levels (Fig. 2), and reduced levels of inflammatory factors (Table 2). On the 8th day of hospitalization, she received radioactive iodine treatment for Graves’ disease. After that, her condition remained stable and she was discharged on the 20th day of hospitalization.

**Table 2:** Changes of inflammatory factor levels before and after 2 courses of DPMAS combined with plasma exchange therapy.

Inflammatory markers	Before treatment	After treatment
Interleukin-2 receptor (U/ml)	>7500*	5050
Interleukin-8 (pg/ml)	76.00	27.5
Interleukin-10 (pg/ml)	54.70	11.1
TNF-α (pg/ml)	50.70	22.10

* This indicator exceeds the upper limit of detectable value.

After discharge, the patient’s outpatient follow-up showed that the symptoms and signs of hyperthyroidism gradually improved, thyroid hormone level gradually decreased, and liver function and blood routine test results remained normal. Hypothyroidism occurred 1 year after radioactive iodine therapy, and her thyroid function returned to normal after taking 25 µg of levothyroxine daily.

## 3. Discussion

Graves’ disease is an autoimmune disease. It accounts for 70% of the causes of hyperthyroidism and occurs in approximately 20 cases per 100,000 people annually.^[5]^ Among ATDs for the treatment of Graves’ disease, methimazole is the first choice because of its long half-life and relatively low hepatotoxicity.^[1]^ PTU has a short half-life, a relatively low rate of transplacental transmission, and can inhibit the conversion of FT4 to FT3 in large doses, making it a treatment option for early pregnancy or cases of thyroid storm.^[6]^ Both can cause liver injury, with the incidence of liver injury caused by methimazole being approximately 1.4% and PTU being approximately 6.4%, respectively.^[7]^ Methimazole-induced liver injury is characterized by cholestatic hepatitis with increased bilirubin level, while PTU-induced liver injury is mainly characterized by hepatocellular hepatitis with elevated transaminase levels. In severe cases, PTU can cause fatal liver necrosis. Because of the overlapping side effects of methimazole and PTU, if a patient is intolerant to one of them, the other should be used with caution. In such situations, radioiodine therapy is strongly recommended. Our patient was highly suspected of methimazole-induced liver injury because of her markedly elevated bilirubin level, normal liver function before taking methimazole, and normal hepatotropic virus and autoimmune hepatitis markers. It’s worth noting that EBV can cause liver injury, which is often subclinical and self-limited.^[8]^ EBV-induced liver injury typically manifests as transient, mild elevations in transaminases. In a few patients, transaminases are significantly elevated, exceeding 5 to 10 times the upper limit of normal.^[9]^ The incidence of EBV-induced jaundice is very low, and acute liver failure can occur in rare cases. Although methimazole was considered the main cause of our patient’s liver injury, EBV infection could not be ruled out as a contributing factor. A liver biopsy may be helpful in clarifying the etiology. However, considering the complex and critical condition of our patient, liver biopsy wasn’t recommended. In view of the above considerations, she immediately stopped taking methimazole and was given ganciclovir treatment for EBV infection.

Thyroid storm, also referred to as thyroid crisis, is a critical condition with a high mortality rate. The Burch Wartofsky Point Scale was developed by Burch and Wartofsky, and is most commonly used for the diagnosis of thyroid storm.^[10]^ This scale is a comprehensive assessment of a patient’s body temperature, cardiovascular condition, gastrointestinal liver function, and mental state. A score >45 points, indicated that the patient had thyroid storm. Our patient was in a state of thyroid storm because her Burch Wartofsky Point Scale score was 80. Drugs for thyroid storm include PTU, propranolol, iodine, and glucocorticoids.^[4]^ As mentioned previously, the patient was not administered PTU because of abnormal liver function. She would undergo radioiodine therapy for Graves’ disease later, and iodine should be discontinued for at least 7 days before that, so it would be best for her not to use iodine. Plasma exchange, an alternative treatment for thyroid storm, was first demonstrated to have beneficial clinical and biochemical effects in patients with thyroid storm in 1970 by Ashkar.^[11]^ Plasma exchange is the process of separating plasma from blood cells by using a plasma separator, discarding plasma containing pathogenic substances and replacing it with fresh plasma or plasma substitutes. It treats a variety of diseases by removing harmful substances from the blood and has a wide range of indications, including severe autoimmune diseases, neurological diseases, some kidney diseases, etc.^[12]^ The vast majority of thyroid hormones are bound to serum proteins in the human body, and the amount of free thyroid hormones is very low.^[13]^ Plasma exchange can effectively remove thyroid hormone bound to serum proteins and rapidly reduce the level of thyroid hormone in the blood.^[14]^ It can also remove some thyroid-related antibodies, immune complexes and inflammatory factors in the blood during thyroid storm.^[15]^ Therefore, in the latest 9th edition of Apheresis Applications Committee of the American Society for Apheresis (ASFA) guideline classifies thyroid storm asⅡcategory indication for plasma exchange.^[12]^ Our patient had a severe thyroid storm with elevated levels of inflammatory factors and thyroid antibodies, so plasma exchange therapy was a good choice for her.

In addition to being in a state of thyroid storm, our patient had obvious liver function abnormalities. DPMAS combined with plasma exchange therapy was used to restore her liver function and control thyroid storm simultaneously. DPMAS is a non-biological artificial liver support system. In DPMAS treatment, the separated plasma passes through the bilirubin adsorption column BS330 to specifically adsorb bilirubin and bile acid, and passes through the disposable hemoperfusion cartridge HA330-II to adsorb inflammatory mediators and toxins.^[16]^ Indications for DPMAS include liver failure, prehepatic failure, severe hyperbilirubinemia, hepatic encephalopathy, severe cholestatic liver disease, perioperative treatment of liver transplantation, and multiple organ dysfunction syndrome with jaundice or sepsis.^[17]^ In the latest 9th edition of ASFA guideline, acute liver failure was classified asⅠcategory indication for plasma exchange, because this treatment can remove toxins while replenishing coagulation factors.^[12]^ As both DPMAS and plasma exchange can improve liver function, DPMAS combined with plasma exchange is clinically used primarily for severe acute liver failure.^[17]^ However, our patient didn’t have acute liver failure, and the primary goal of plasma exchange therapy was to control her thyroid storm. Compared to DPMAS alone or plasma exchange alone, DPMAS combined with plasma exchange therapy can not only quickly improve liver function, especially hyperbilirubinemia, but also increase the clearance rate of thyroid hormones, toxins, immune complexes and inflammatory factors. This combined therapy can also supplement coagulation factors, albumin, and other blood substances consumed by the simple DPMAS therapy. Our patient’s rapid recovery was the best evidence for the efficacy of this combination therapy.

Radioactive iodine therapy can cause thyroiditis, leading to a transient increase in the release of thyroid hormones.^[18]^ Therefore, patients with Graves’ disease are at risk of transient worsening of their condition after radioactive iodine therapy. Choosing the right time to receive radioactive iodine therapy is very important for patients with Graves’ disease who are in critical condition, such as our patient. A previous study found that in patients with hyperthyroidism and abnormal liver function, liver function indicators remained at a low level 3 days after the artificial liver support system and began to rebound 4 to 7 days later. Therefore, this study recommended timely radioactive iodine treatment within 3 days of artificial liver treatment.^[19]^ In our patient, when her bilirubin and thyroid hormone levels decreased significantly after 2 courses of DPMAS combined with plasma exchange therapy, radioactive iodine therapy was performed immediately. Choosing the right time for radioactive iodine therapy was also key to quick recovery of our patient.

## 4. Conclusion

In this paper, we report a case of Graves’ disease patient treated with methimazole who developed severe liver injury, mononucleosis, EBV infection, and thyroid storm. Her condition was extremely critical, but greatly improved after DPMAS combined with plasma exchange therapy and radioactive iodine treatment. This suggests that plasma exchange is an effective treatment for thyroid storm in addition to conventional drug therapy, and DPMAS combined with plasma exchange therapy is a good choice for patients with thyroid storm and severe liver injury.

## Author contributions

**Data curation:** Jiali Liu, Weiwei Zhang.

**Investigation:** Weiwei Zhang.

**Software:** Jiali Liu.

**Writing – original draft:** Jiali Liu, Weiwei Zhang.

**Writing – review & editing:** Weiwei Zhang.
